# Research Progress and Applications of the Rotavirus Reverse Genetics System

**DOI:** 10.3390/ani16040608

**Published:** 2026-02-14

**Authors:** Yiqun Chen, Jie Chen, Tao Li, Mingyu Fan, Jun Li, Jing Wang, Zengwen Huang, Jingang Zhao, Chaoyun Yang, Zhiqiang Hu

**Affiliations:** 1 College of Animal Science, Xichang University, Xichang 615000, China; xcc20250039@xcc.edu.cn (Y.C.); 17311729594@163.com (T.L.); lijun@xcc.edu.cn (J.L.); slnee.5703@163.com (J.W.); xndaxue@126.com (Z.H.); zjingang2022@163.com (J.Z.); chaoyuny@yeah.net (C.Y.); 2 College of Veterinary Medicine, South China Agricultural University, Guangzhou 510642, China; 3062351174@stu.scau.edu.cn; 3 College of Veterinary Medicine, Northwest A & F University, Yangling 712100, China; keepitup9968@126.com; 4 Key Laboratory of Animal Epidemic Disease Detection and Prevention in Panxi District, College of Animal Science, Xichang University, Xichang 615013, China

**Keywords:** rotavirus, double-stranded RNA virus, reverse genetics, vaccine development

## Abstract

Rotavirus is a leading cause of severe, dehydrating diarrhea in infants and young animals, causing significant global morbidity and mortality. For decades, research was hindered by challenges in establishing reverse genetics systems due to the virus’s complex segmented genome and poor cell culture adaptation. The first helper virus-dependent system (2006) was limited by low efficiency. A 2017 breakthrough established the first fully plasmid-based system, which eliminated helper viruses and revolutionized the field. Subsequent optimizations, such as codon modification and CRISPR/Cas9 integration, have significantly enhanced efficiency, enabling viable systems for diverse human and animal strains. This narrative review summarizes the evolution and technological milestones of rotavirus reverse genetics. We discuss critical applications in analyzing viral gene function, developing novel vaccines, screening antiviral drugs, and investigating cross-species transmission. Finally, we provide an outlook on the future prospects of this transformative technology.

## Simple Summary

Rotavirus (RV) remains a leading cause of severe, dehydrating diarrhea in infants and young animals, imposing significant global morbidity, mortality, and economic burdens. Historically, the development of effective interventions has been impeded by the technical challenges inherent to manipulating its complex, segmented RNA genome. This review highlights pivotal advancements in rotavirus reverse genetics—a technology enabling the de novo generation of infectious virus entirely from cloned cDNA. Transitioning from early, inefficient systems, this technology has matured into a viable and versatile platform, now facilitating the precise genetic engineering of diverse rotavirus strains from both human and animal hosts. These capabilities are instrumental for elucidating viral pathogenesis, designing next-generation vaccines, establishing high-throughput platforms for antiviral drug discovery, and investigating transmission dynamics. Collectively, these advances provide essential tools to accelerate the development of improved interventions to mitigate the global burden of rotavirus disease.

## 1. Introduction

Rotavirus (RV), a member of the genus Rotavirus within the family Reoviridae, possesses a genome of approximately 18.5 kb. This genome comprises 11 segments of double-stranded RNA (dsRNA) encoding six structural proteins (VP1–VP4, VP6, VP7) and 5–6 non-structural proteins (NSP1–NSP5/6) [1]. The viral particle features a triple-layered capsid architecture: the inner core, formed by VP1–VP3, orchestrates genome replication and transcription; the middle capsid, composed of VP6, serves as the group-specific antigenic determinant; and the outer capsid, comprising VP4 and VP7, constitutes the primary neutralizing antigens and protective immunogens [2,3]. According to the 2022 taxonomy by the International Committee on Taxonomy of Viruses (ICTV), rotaviruses are classified into nine species (A–D and F–J) based on the antigenic properties of VP6 [4]. Among these, Rotavirus A (RVA) stands out as the primary etiological agent of acute diarrhea in human infants and young children, while also infecting a diverse array of animal hosts, including swine, cattle, and poultry. The dual classification system based on the outer capsid proteins VP7 (G type) and VP4 (P type) highlights the extensive genetic diversity of the virus. To date, 42 G genotypes and 58 P genotypes have been identified, a diversity that underpins the virus’s sustained prevalence and capacity for immune evasion [5].

RVA poses a persistent threat across both public health and veterinary sectors. In humans, it accounts for over 100,000 annual pediatric deaths worldwide [6]. In agriculture, Porcine Rotavirus devastates piglet populations in China with high morbidity (50–80%) and mortality (10–50%), complicated by a recent genotypic shift from G5 to G9 [7,8,9]. Similarly, Bovine Rotavirus is a primary pathogen responsible for neonatal calf diarrhea, severely impacting the cattle industry. It typically afflicts calves aged 1–3 weeks, manifesting as severe watery diarrhea, dehydration, and depression [10,11]. Avian Rotavirus induces enteritis in poultry, such as chickens and turkeys, causing growth retardation and a 15–30% reduction in feed conversion efficiency, thereby compromising the economic viability of poultry farming [12,13]. Furthermore, the potential for cross-species transmission and genetic reassortment underscores the zoonotic risk of rotaviruses. Consequently, intensified research into the virological characteristics and transmission dynamics of rotavirus is critical for the prevention and control of zoonoses and the development of effective vaccines.

Reverse genetics, which enables the targeted manipulation of viral genomes to elucidate the relationship from genotype to phenotype, stands as a cornerstone technology in modern virology [14]. This strategy has been instrumental in advancing the study of diverse RNA viruses, including coronaviruses, influenza viruses, and flaviviruses [15,16,17,18,19]. In contrast, the establishment of reverse genetics systems for rotavirus has historically lagged behind, a delay that has severely impeded functional genomics research and the development of novel vaccine vectors.

By contrast, the establishment of reverse genetics systems for rotavirus has progressed through a series of discrete, landmark stages over more than a decade, reflecting virus-specific biological constraints.

Within the Reoviridae family, reverse genetics systems have been successfully established for several segmented double-stranded RNA viruses, including Mammalian orthoreovirus [20], Nelson bay Orthoreovirus [21], Bluetongue virus [22], African horse sickness virus [23], and Epizootic hemorrhagic disease virus [24]. These systems, largely developed between the mid-2000s and early 2010s, enabled efficient recovery of infectious virus using RNA- or plasmid-based approaches in conventional cell lines, thereby defining early technical benchmarks for segmented dsRNA viruses.

In contrast, rotavirus reverse genetics remained refractory until 2006, when the first helper virus-based segment replacement system was reported, and did not achieve a fully plasmid-based rescue until 2017. Subsequent studies have focused on systematic optimization of rescue efficiency and strain coverage, underscoring a protracted and stepwise developmental trajectory distinct from that of other Reoviridae members. In this narrative review, we focus on representative and landmark studies that have shaped the development of rotavirus reverse genetics. Studies were selected based on their introduction of major technical milestones, significant improvements in rescue efficiency or system simplification, and/or their subsequent widespread adoption and influence in the field. This approach aims to provide a coherent overview of the stepwise evolution and current maturity of rotavirus reverse genetics systems.

## 2. Advances in the Study of Rotavirus Reverse Genetics Systems

Over the past two decades, the field of rotavirus reverse genetics has undergone a remarkable evolution. Transitioning from initial helper virus-dependent proof-of-concept studies to the establishment of fully plasmid-based systems, the technology has recently advanced through systematic optimization for diverse strains and research contexts. Today, this methodological framework has reached a stage of maturity.

### 2.1. Early Exploration of Reverse Genetics Systems Relying on Helper Viruses

For decades, the establishment of a stable and efficient genetic manipulation system remained elusive, largely due to the technical hurdles imposed by the complex segmented double-stranded RNA genome structure. A milestone was achieved in 2006 with the development of the first helper virus-dependent reverse genetics system for rotavirus, a breakthrough that laid the foundation for research in this field [25]. This protocol employed the attenuated recombinant vaccinia virus rDIs-T7pol to drive T7 RNA polymerase expression in SV40-transformed African green monkey kidney cells (COS-7). Experimental procedures involved the transfection of a plasmid encoding the VP4 gene of the monkey rotavirus SA11-L2 strain—flanked by a T7 promoter at the 5′ end and a T7 terminator and hepatitis delta virus ribozyme sequence at the 3′ end—followed by superinfection with the authentic human group A rotavirus (strain KU) as a helper virus. Following serial passage in MA104 cells, a recombinant virus rKU carrying the SA11-L2 VP4 gene was successfully rescued.

Subsequently, Troupin et al. [26] and Trask et al. [27] further refined the rescue efficiency and purification feasibility of recombinant viruses by optimizing transfection protocols and employing novel strategies such as temperature-sensitive mutants and dual selection mechanisms. Collectively, these findings provided critical proof of concept that rotavirus genome fragments can be directionally replaced and stably passaged, thereby establishing the theoretical foundation for the development of fully plasmid-based reverse genetics systems.

Despite these conceptual advances, helper virus-dependent systems remained constrained by intrinsic limitations: first, the cytopathic effect (CPE) induced by vaccinia virus replication may lead to cell lysis before the production of recombinant viruses; second, laborious screening protocols—such as neutralizing antibodies, host range restriction, or temperature selection—were necessitated to purify the recombinant virus; and third, the experimental protocol was technically demanding, time-consuming, and suffered from low rescue efficiency [28]. Consequently, establishing a helper-virus-free, fully plasmid-based reverse genetic system emerged as the primary objective in this field.

### 2.2. Establishment of a Plasmid-Based Rotavirus Reverse Genetics System

**Figure 1:**
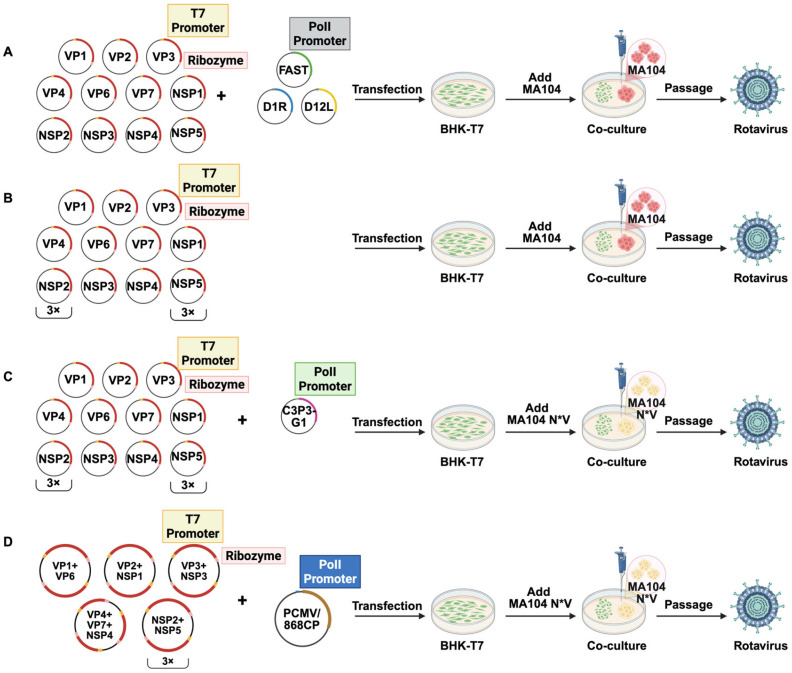
Schematic of the plasmid-based reverse genetics system for rotavirus. (**A**) 14-plasmid reverse genetics system. Different colors on the plasmid circles are used solely to distinguish different plasmids; (**B**) 11-plasmid reverse genetics system. “3×” indicates a threefold transfection amount. MA104 N*V cells are derived from MA104 cells (monkey embryonic kidney cells) and co-express the V protein of parainfluenza virus type 5 (PIV5) and the N protein of bovine viral diarrhea virus (BVDV). The V protein targets STAT1 for degradation, while the N protein targets IRF3 for degradation. (**C**) 12-plasmid reverse genetics system; (**D**) 5-plasmid reverse genetics system.

A pivotal milestone in rotavirus research was the development of the first fully plasmid-based reverse genetics system in 2017 [29]. This revolutionary approach entailed the cloning of all 11 gene segments of the rotavirus SA11-L2 strain into individual plasmids, flanked by a 5′ T7 promoter and a 3′ cassette containing a T7 terminator and a hepatitis D virus ribozyme. These 11 viral plasmids were subsequently co-transfected into BHK-T7 cells (which stably express T7 RNA polymerase) supplemented with three essential helper plasmids. Cell lysates, harvested via freeze-thaw cycles, were passaged in MA104 cells. Successful viral rescue was evidenced by the observation of pronounced CPE several days later [29]. The efficacy of this system relied on the co-transfection of three helper plasmids supplying essential functions *in trans*. The first plasmid expressed the Nelson Bay virus Fusion-Associated Small Transmembrane (FAST) protein, a fusogen that mediates cell-cell fusion and syncytium formation. This mechanism facilitates rapid intercellular viral dissemination and enhances the release of progeny virions via syncytial lysis [30,31]. The remaining two helper plasmids encoded the dual subunits (D1R and D12L) of the vaccinia virus capping enzyme. This enzyme catalyzes the addition of a 5′ cap structure to the nascent rotavirus mRNA transcripts, a modification critical for protecting them from exonuclease degradation and for ensuring their efficient translation [32]. A schematic of this 14-plasmid reverse genetics protocol is illustrated in Figure 1A.

This breakthrough revolutionized the technological landscape of rotavirus functional genomics research, enabling site-directed mutagenesis, gene deletion, and exogenous gene insertion, while serving as a universal platform for the development of attenuated vaccines and viral vectors.

### 2.3. Systematic Optimization Strategy for Whole Plasmid Reverse Genetics System

Following the establishment of the fully plasmid-based system, multiple research teams have continuously optimized the platform around three core objectives: simplifying the helper plasmid system, enhancing transfection efficiency, and expanding the applicability to diverse strains (summarized in Table 1).

Firstly, regarding system simplification, investigations revealed that certain helper plasmids are dispensable for virus rescue. Komoto et al. [33,34] demonstrated that the overexpression of NSP2 and NSP5 significantly enhances rescue efficiency, thereby simplifying the system to an 11-plasmid system. This strategy highlights the pivotal role of viroplasm formation in rotavirus replication and assembly [35] and has since become a widely adopted basic platform in subsequent investigations. A schematic of the resulting 11-plasmid reverse genetics operation procedure is illustrated in Figure 1B.

Secondly, to address the substantial disparity in rescue efficiency between human and animal rotaviruses [36]. Komoto et al. [37], optimized the 11-plasmid system to successfully rescue the human rotavirus KU strain. Key strategies included elevating the trypsin concentration in the culture medium, implementing co-culture of BHK-T7 and CV-1 cells, rolling-tube incubation, and plaque purification. This study established a foundational framework for the reverse genetics of human rotavirus.

Concurrently, the supplementation of key non-structural proteins via eukaryotic expression plasmids, the engineering of host cell lines with suppressed interferon (IFN) signaling pathways, and the utilization of highly efficient viral capping enzymes have significantly enhanced the system’s versatility and rescue efficiency across different strains. In 2020, Kobayashi et al. [38] established a fully plasmid-based reverse genetic system for the first time by supplementing the expression of NSP2 and NSP5. Utilizing this system to analyze the effect of an NSP1 C-terminal deletion on viral replication, results indicated that the function of degrading host β-TrCP is dispensable in the cell line. Subsequently, Sanchez-Tacuba et al. [39] engineered the MA104 cell line with a suppressed IFN response. By combining this with an anti-IFN strategy targeting Signal Transducer and Activator of Transcription 1 (STAT1), and IFN Regulatory Factor 3 (IRF3) [40,41] and a highly efficient NP868R capping enzyme helper plasmid [42], they successfully rescued a variety of human and animal rotavirus strains, significantly expanding the applicability of the system. However, its broad utility may still be limited by strain-dependent variations in IFN antagonism. A schematic of the resulting 12-plasmid reverse genetics operation protocol is illustrated in Figure 1C.

Finally, in recent years, the optimization of reverse genetic systems has gradually shifted from the “operational level” to the “molecular and host level.” Codon optimization of NSP2 and NSP5 genes [43], CRISPR/Cas9 screening for host restriction factors [44], and optimization of T7 RNA polymerase-high expression cell lines [45] have all significantly reduced the dependence on transfection conditions and plasmid quantity, and for the first time achieved stable rescue of multiple clinical isolates. These advances have solved key technical problems that have long restricted the molecular epidemiology and vaccine strain research of rotavirus.

It is noteworthy that the introduction of plasmid integration strategies represents a new direction in the development of reverse genetic systems. Building on this precedent, researchers integrated the rotavirus 11 plasmid system into a 5-plasmid system for the first time, successfully rescuing the sheep rotavirus vaccine strain LLR [46]. This strategy improves the stability, reproducibility, and scalability of the system by reducing the complexity of plasmid co-transfection, and has important application potential, especially suitable for the rapid construction and evaluation of veterinary vaccine strains. A schematic of the resulting 5-plasmid reverse genetics operation procedure is illustrated in Figure 1D.

**Table 1:** Summary of optimization strategies and key innovations in rotavirus reverse genetics.

Strain	Host Species	No. of Plasmids	Auxiliary Factors	Optimization Strategy	Key Outcomes	References
SA11	Simian	14	FAST; VV capped enzyme	Not applicable	First fully plasmid-based rotavirus reverse genetics system	[29]
SA11	Simian	11	NSP2; NSP5	Reduction in helper plasmid number	Markedly improved virus rescue efficiency	[34]
KU	Human	11	NSP2; NSP5	Increased pancreatic enzyme concentration	First successful rescue of a human rotavirus strain	[37]
Odelia	Human	16	FAST; VV capped enzyme; NSP2; NSP5	Addition of eukaryotic expression plasmids (pCAG-NSP2/NSP5)	Expanded applicability to human, simian, and murine strains	[38]
RRV	Simian	12	ASFV NP868R capped enzyme; NSP2; NSP5	Addition of C3P3-G1 helper plasmid and modified MA104 cell line		[39]
SA11	Simian					
CDC-9	Human					
D6/2	Murine-like					
D6/2-2g	Murine	12	C3P3-G3; NSP2; NSP5	Introduction of next-generation helper plasmid C3P3-G3 and SERPINB1-knockout MA104 cells	Reduced plasmid number and transfection reagent requirements	[44]
C73, HM26, BLR	Bovine	11	NSP2; NSP5	Establishment of BHK-T7-derived BHK-M3 cells by flow cytometric selection	Enabled reverse genetics systems for bovine rotaviruses	[45]
LLR	Ovine	5	NSP2; NSP5	Reduction in plasmid transfection number	Improved rescue stability and reproducibility	[46]

### 2.4. Expanded Applications of Reverse Genetics Systems for Rotaviruses of Different Species

Driven by the continuous optimization of reverse genetics platforms, the utility of these systems has extended beyond classic laboratory strains to encompass a broad spectrum of animal-derived rotaviruses.

#### 2.4.1. Establishment of a Reverse Genetics System for Porcine Rotavirus

The first fully plasmid-based reverse genetics system for the porcine rotavirus OSU strain was established in 2024 [47]. The development followed a systematic, multi-step workflow, initiating with the complete genome sequencing of the OSU strain via third-generation nanopore technology. Guided by this sequence, a full set of 11 transcription plasmids was constructed. The functionality of each plasmid was individually validated through the generation of single-gene reassortant viruses, utilizing the well-characterized, laboratory-adapted SA11 strain as a genetic backbone.

For the rescue of the authentic virus, an optimized protocol was employed. The complete recombinant OSU virus (rOSU) was successfully recovered by co-transfecting the 11 OSU plasmids supplemented with a helper plasmid expressing the African swine fever virus NP868R capping enzyme. The protocol was further refined by adjusting plasmid stoichiometry to increase the expression of NSP2 and NSP5.

The utility of this platform for genetic engineering was demonstrated by the generation of a derivative virus rOSU-2A-UnaG. This recombinant virus was engineered to express the fluorescent protein UnaG, demonstrating the system’s capacity for introducing and expressing foreign genetic material within the viral genome.

#### 2.4.2. Avian Rotavirus Reverse Genetics System

The application of reverse genetics was extended to avian rotaviruses in 2022 with the establishment of the first fully plasmid-based reverse genetics system for the pigeon PO-13 strain [48]. This advancement opened new avenues for investigating avian rotavirus biology, pathogenesis, and evolution. The study highlighted that the efficient recovery of challenging viral strains can be facilitated by the systematic optimization of key experimental parameters, including promoter selection, plasmid stoichiometry, and the utilization of highly permissive cell lines.

#### 2.4.3. Expansion to Other Key Rotavirus Species

Beyond the porcine and avian systems, reverse genetics platforms have been developed for a broad spectrum of other significant rotavirus strains. Notable examples include key bovine strains (NCDV, UK, and RF) [43,45], the ovine LLR vaccine strain [49], the murine D6/2-2g strain [39], and the prototypic simian laboratory strains SA11 and RRV [29,39]. The availability of these species-specific systems has substantially expanded the experimental scope of the field, providing critical tools to investigate viral evolution, host range restriction, and the molecular determinants of cross-species transmission.

## 3. Application of the Rotavirus Reverse Genetics System

With the maturation of the reverse genetics system, its applications have expanded from initial proof-of-concept validation to encompass diverse research domains, including viral gene function analysis, rational vaccine design, antiviral drug screening, and in vivo infection dynamics. Consequently, it has established itself as a cornerstone platform for modern rotavirus research.

### 3.1. Analysis of Viral Gene Function and Virus-Host Interaction

By employing strategies such as site-directed mutagenesis, gene deletion, or tag insertion, the reverse genetics system facilitates the direct dissection of the contribution of individual genes to viral replication, pathogenicity, and immune escape in the context of a complete virus, thereby substantially enhancing the physiological relevance of research outcomes.

#### 3.1.1. VP3: The Integration Hub of mRNA Modification and Multi-Level Immune Escape

VP3 functions as a core structural protein of rotavirus, acting not only as an mRNA capping enzyme but also as a potent immune regulator [50]. Recent investigations leveraging the reverse genetics system have elucidated that VP3 is multifunctional, serving as a key immune escape factor that subverts multiple host antiviral pathways. Specifically, VP3 exhibits 2′-5′ phosphodiesterase (PDE) activity that degrades 2′-5′ oligoadenylates (2-5A), thereby antagonizing the OAS-RNase L pathway [51]. Experiments utilizing PDE-deficient mutant viruses have conclusively demonstrated that this activity is critical for viral replication and shedding in vivo [52].

Furthermore, VP3 suppresses type I IFN (IFN-I) production by inducing MAVS degradation [53,54], and impairs IFN-stimulated gene (ISG) expression by interfering with the prefoldin complex [55]. Collectively, these findings underscore the role of VP3 as a multifunctional bridge between viral RNA metabolism and host immune evasion, exemplifying the sophisticated strategy of rotavirus to achieve multilayered immune regulation via a single protein.

#### 3.1.2. NSP1: A Key Factor Determining Tissue Tropism and Extraintestinal Pathogenicity

NSP1 has long been recognized as the primary IFN antagonist of rotavirus; however, its specific contribution to in vivo pathogenicity has only recently been elucidated. Utilizing a reverse genetics system, recombinant viruses harboring NSP1 derived from diverse origins were engineered. Results demonstrated that NSP1 serves as a critical determinant of rotavirus extraintestinal replication and the severity of systemic disease [56]. Notably, NSP1 derived from the RRV strain significantly enhances viral replication in extraintestinal tissues and induces severe biliary tract disease, an effect distinct from its classic IRF3 binding domain. This study establishes the pivotal role of NSP1 as a determinant of rotavirus tissue tropism in an in vivo context, providing a molecular basis for understanding rotavirus-associated biliary pathology.

#### 3.1.3. NSP2 and NSP5: Core Modules of Viroplasm Dynamic Regulation

The viroplasm serves as the central hub for rotavirus genome replication and assembly, with NSP2 and NSP5 forming its structural and functional core [35]. Investigations utilizing a reverse genetics system have demonstrated that the post-translational modification status of these two proteins exerts a critical influence on viroplasm assembly, dynamics, and viral replication efficiency [57,58].

Specifically, the phosphorylation state of NSP2 modulates the kinetics of viroplasm assembly and impacts viral yield [57], while the hyperphosphorylation of NSP5 and its C-terminal structure are prerequisites for functional viroplasm formation [58]. Collectively, these studies reveal at the molecular level that the viroplasm is not a static structure, but a dynamically regulated replication factory.

#### 3.1.4. NSP4: The Association Between Glycosylation Modification and Replication Efficiency and Pathogenicity

The rotavirus NSP4 protein functions as an endoplasmic reticulum transmembrane glycoprotein characterized by two N-glycosylation sites that remain conserved across diverse host species. Utilizing glycosylation-deficient recombinant viruses, investigations have demonstrated that the N-glycosylation of NSP4 exerts a critical regulatory impact on viral replication efficiency and in vivo pathogenicity [59]. These findings imply that post-translational modifications of NSP4 not only modulate viral replication but may also contribute to host-specific adaptation processes.

### 3.2. Novel Vaccine Design and Development

Reverse genetics serves as a pivotal platform for the rational design and rapid evaluation of rotavirus vaccine candidates, accelerating the transition of vaccine development from empirical screening to mechanism-driven strategies.

#### 3.2.1. Genotype Matching and Analysis of Antigenic Determinants

By substituting the VP4 or VP7 gene within a common genetic backbone, researchers can systematically evaluate the susceptibility of diverse prevalent genotypes to neutralizing antibodies [60,61]. In 2020, Kobayashi et al. [60] utilized the simian rotavirus SA11 backbone to incorporate VP4 and VP7 genes derived from human clinical isolates, thereby rapidly generating recombinant vaccine candidates representing distinct G and P genotypes. The study revealed that the replication competence of recombinant viruses harboring heterologous VP4 genes was significantly attenuated, indicating that VP4 serves as a critical determinant of viral infectivity. This approach facilitates the rapid evaluation of candidate vaccine strains matching circulating genotypes, thereby substantially accelerating the development cycle compared to traditional reassortment screening.

In 2025, Kotaki et al. [61] leveraged the reverse genetics system of the human rotavirus Odelia strain to construct a mono-reassortant virus library via the substitution of VP4 or VP7 segments, covering 7 VP4 and 17 VP7 genotypes. They systematically assessed the susceptibility of these genotypes to neutralizing antibodies elicited by vaccination or natural infection. These studies elucidated that VP7, rather than VP4, functions as the dominant determinant of neutralization susceptibility. Furthermore, they mapped key antigenic domains, providing a theoretical framework for predicting the evolutionary trends of circulating strains and optimizing vaccine antigen selection.

#### 3.2.2. Single-Round Infectious Rotavirus: A New Generation of Safety-Oriented Vaccine Platforms

In recent years, single-round infectious rotaviruses engineered via reverse genetics systems have emerged as a promising avenue in vaccine research [61,62]. These viruses are restricted to a single replication cycle in wild-type cells, thereby significantly mitigating the risk of reversion to virulence while preserving native immunogenicity. This strategy offers a new paradigm for developing oral vaccines and viral vectors characterized by both high immunogenicity and an enhanced safety profile.

#### 3.2.3. Rotavirus as a Multivalent Vaccine Expression Vector

Rotavirus offers distinct advantages as a vector for oral vaccines targeting intestinal pathogens. Key features include a preferential tropism for the small intestine, which facilitates antigen delivery to key mucosal sites; an acute, self-limiting infection profile devoid of genomic integration; high intrinsic immunogenicity capable of eliciting robust systemic and mucosal immunity; and a segmented genome that is highly amenable to genetic manipulation.

Owing to its specific intestinal tropism and capacity to elicit robust mucosal immunity, rotavirus has been extensively explored as a vector for the expression of heterologous antigens [63,64,65,66]. Through the insertion of exogenous antigens into non-structural protein genes, candidate combination vaccines against SARS-CoV-2 and human norovirus have been successfully engineered. These achievements validate the utility of the rotavirus platform for developing multivalent intestinal vaccines.

### 3.3. Development of Antiviral Drug Screening and Immunological Assessment Tools

The integration of fluorescent or luminescent reporter genes into the rotavirus genome renders it a robust platform for high-throughput antiviral drug screening and neutralizing antibody detection. These reporter virus systems not only enhance screening efficiency but also offer a standardized approach for cross-species immunogenicity evaluation. For instance, researchers engineered a genetically stable bovine rotavirus NSP3-ZsGreen reporter virus to successfully screen 12 potential antiviral drugs, thereby validating the feasibility of high-throughput drug screening [45]. Additionally, researchers established a high-throughput micro-neutralization assay utilizing the SA11-GFP reporter virus. This method bypasses the reliance on species-specific secondary antibodies, providing a rapid, standardized, and universal tool for evaluating vaccine immunogenicity across diverse animal models [67].

Building on this strategy, a reporter virus was generated by inserting the GFP gene into the SA11 backbone. The resulting construct facilitated the development of a micro-neutralization assay that quantifies antibody activity by measuring the reduction in fluorescence. A distinct advantage of this system is its species-independent nature; it obviates the need for species-specific secondary antibodies that are prerequisites for traditional methods like ELISA. Consequently, this technology allows for the rapid and standardized assessment of neutralizing antibody titers in serum from diverse animal species, positioning it as a powerful and versatile tool for preclinical and clinical vaccine evaluation.

### 3.4. In Vivo Imaging and Viral Transmission Dynamics Studies

The integration of reverse genetics systems with in vivo imaging technology enables the real-time, non-invasive monitoring of rotavirus replication, transmission, and tissue tropism within the living host. In 2022, Zhu et al. [68] engineered the first recombinant mouse rotavirus (mRV-NLuc) expressing the NanoLuc luciferase. Using the IVIS in vivo imaging system, they observed that robust luminescent signals were localized to the small intestine (proximal and distal) and colon of mice following infection. In vivo imaging revealed that viral replication peaked 2–5 days post-infection. Notably, luminescent signals were detectable in cohabiting mice as early as 3 days post-exposure, preceding the onset of clinical diarrhea and fecal viral shedding. In STAT1-/- mice, luminescence levels were significantly elevated. This system provides a novel, non-invasive, dynamic, and quantitative platform to assess rotavirus infection, tissue tropism, and the impact of host factors, offering a valuable tool for vaccine and antiviral intervention research.

## 4. Discussion

The establishment and optimization of the rotavirus reverse genetics system represent a pivotal milestone in molecular virology. Its evolution from the initial helper-dependent proof-of-concept (2006) to the fully plasmid-based platform (2017) has solidified its status as a cornerstone of rotavirus research, propelling both fundamental and translational advances.

Currently, the system demonstrates substantial utility across key domains. In pathogenesis, it facilitates the genetic dissection of proteins like VP3 and NSP1, elucidating their roles in immune evasion and tissue tropism. In vaccinology, it enables the rapid generation of genotype-matched candidates and next-generation platforms (e.g., Single-Round Infectious Rotavirus). Furthermore, reporter-expressing viruses have established robust platforms for high-throughput antiviral screening.

Despite these advancements, challenges persist. Primary constraints include suboptimal rescue efficiency for certain clinical or non-canonical strains and the genetic instability of large insertions (>1 kb). Moreover, disparities between in vitro and in vivo performance often necessitate further optimization. Finally, the taxonomic scope remains limited; expanding efficient systems to other epidemiologically significant animal strains—such as equine and ovine rotaviruses—remains a critical priority.

## 5. Conclusions

Although rotavirus reverse genetics platforms have reached a high level of technical sophistication, their practical accessibility remains uneven. At present, efficient and reproducible virus rescue is largely confined to laboratories with dedicated experience in reverse genetics. Further simplification and standardization of these systems will be critical for facilitating broader adoption and maximizing their impact across the field.

## 6. Future Perspectives

Having evolved from initial proof of concept to a mature methodology over the past two decades, the rotavirus reverse genetics system has transitioned from a focus on technical optimization to application-driven inquiries. Moving forward, the utility of this system will be measured by its capacity to elucidate fundamental biological questions and address unmet public health challenges.

From a technological perspective, the next frontier prioritizes enhancing precision and accessibility. The convergence of CRISPR-based editing tools and AI-driven structural prediction (e.g., AlphaFold) will facilitate a high-throughput “prediction-validation” loop for functional motif analysis. Simultaneously, the development of simplified plasmid systems and standardized rescue cell lines will broaden the accessibility of the technology, enabling its widespread application across diverse research settings.

Regarding model systems, integrating reverse genetics with physiologically relevant models—such as human intestinal organoids and microfluidic organ-on-a-chip platforms—coupled with spatial multi-omics, offers a unique opportunity to recapitulate the complex host-pathogen interface. This multidimensional approach is essential for dissecting the intricate molecular mechanisms of rotavirus pathogenesis.

In terms of translational potential, the platform holds transformative promise for vaccine development. Given the continuous antigenic drift of circulating strains, reverse genetics provides a versatile platform for the rapid generation of genotype-matched vaccine candidates. In particular, the engineering of single-round infectious particles and multivalent vectors represents a promising strategy for developing safer, more effective interventions.

In summary, the rotavirus reverse genetics system is transitioning from a specialized tool to a comprehensive platform for translational virology. As this technology matures, it is poised to assume a pivotal role in the global effort to understand, prevent, and control rotavirus disease.

## Data Availability

No new data were generated in this study. All data discussed are derived from previously published studies cited in the manuscript.
